# Applied Rheology as Tool for the Assessment of Chitosan Hydrogels for Regenerative Medicine

**DOI:** 10.3390/polym13132189

**Published:** 2021-06-30

**Authors:** Pablo Sánchez-Cid, Mercedes Jiménez-Rosado, María Alonso-González, Alberto Romero, Victor Perez-Puyana

**Affiliations:** 1 Department of Chemical Engineering, Faculty of Chemistry, Universidad de Sevilla, 41012 Sevilla, Spain; pscb14495@yahoo.es (P.S.-C.); alromero@us.es (A.R.); vperez11@us.es (V.P.-P.); 2 Department of Chemical Engineering, Higher Polytechnic School, Universidad de Sevilla, 41012 Sevilla, Spain; maralonso@us.es

**Keywords:** chitosan, hydrogel, soft tissue, regenerative medicine, pH change, thermal resistance, rheology

## Abstract

The regeneration of soft tissues that connect, support or surround other tissues is of great interest. In this sense, hydrogels have great potential as scaffolds for their regeneration. Among the different raw materials, chitosan stands out for being highly biocompatible, which, together with its biodegradability and structure, makes it a great alternative for the manufacture of hydrogels. Therefore, the aim of this work was to develop and characterize chitosan hydrogels. To this end, the most important parameters of their processing, i.e., agitation time, pH, gelation temperature and concentration of the biopolymer used were rheologically evaluated. The results show that the agitation time does not have a significant influence on hydrogels, whereas a change in pH (from 3.2 to 7) is a key factor for their formation. Furthermore, a low gelation temperature (4 °C) favors the formation of the hydrogel, showing better mechanical properties. Finally, there is a percentage of biopolymer saturation, from which the properties of the hydrogels are not further improved (1.5 wt.%). This work addresses the development of hydrogels with high thermal resistance, which allows their use as scaffolds without damaging their mechanical properties.

## 1. Introduction

Tissue engineering (TE) is based on the application of principles and methods from engineering and life sciences through the fundamental comprehension of the relationship between structure and function in normal and pathologic tissues and the development of biological substitutes to restore, maintain or enhance their functions [1]. To this end, TE combines three key components: cells, growth factors and scaffolds [2].

Among scaffolds, hydrogels have gained considerable interest and attention in TE research, due to their compositional and structural similarities with native tissues, as well as their suitability for cell proliferation and survival. Hydrogels are tridimensional networks of hydrophilic reticular polymers bound covalently, and through intra- or inter-molecular physical attractions. They can absorb up to 1000% of their dry weight in water. Their most remarkable properties are hydrophilicity, insolubility in water, elasticity and softness [3,4]. However, as biomedical materials, hydrogels require severe sterilization before use and must be able to withstand body temperatures once introduced into the organism in order to prevent decomposition [5]. Thus, thermal stability is a very important characteristic to consider in the development of hydrogels, to select the raw materials and processing conditions properly.

Regardless of the application, selecting an adequate raw material for the production of scaffolds with an optimal internal structure is highly beneficial for cell activity. In this way, scaffolds can be made from natural or synthetic polymers. It is important to point out that synthetic materials are the most used nowadays, due to the ease of controlling and modifying the final properties of the scaffolds. Nevertheless, most of these materials lead to certain problems related to immunogenicity and inflammation once introduced into the body [6]. To overcome these drawbacks, natural materials have been presented as potential candidates to be used in the fabrication of scaffolds for TE [7]. Biopolymers are increasingly used in TE due to their structure and properties, where low antigenicity and inflammation are combined with high hydrophilicity, good cytotoxic response, biocompatibility, biodegradability and mucoadhesiveness; however, low mechanical and structural properties hinder the application of these biopolymers [8].

The most popular biopolymers are proteins and polysaccharides [9,10,11,12]. Polysaccharides are biopolymers composed of a wide succession of monosaccharides or disaccharides linked repeatedly by O-glycosidic bonds. They fulfill different functions in the organism, namely, contributing to the development of organic structures and energy storage and they can also act as a protection mechanism [13]. Among the different polysaccharides, the most used for scaffold fabrication are starch, alginate, chitin/chitosan, hyaluronic acid and their derivatives [14,15]. The presence of amino groups in the polymeric chain makes chitosan one of the most versatile materials due to the possibility of performing a wide variety of modifications on its properties and structure. The degradation rate, physico-chemical and biological properties of chitosan depend on its deacetylation degree and its molecular weight. It has been described as a linear cationic, biodegradable and environmentally friendly polymer [16]. Nowadays, research on chitosan hydrogels has gained attention for many applications, such as high-performance lubricants [17], regenerative medicine [18] and controlled delivery of pharmaceuticals, the latter being focused on hydrogel composition modification and drug release kinetics. The composition modification strategies include, for example, the application and/or combination of different biopolymers and the addition of enzymes in order to modify not only the composition of the resulting hydrogel, but also its surface properties [19,20]; however, thermal instability at body temperature and the variable pH environment cause poor drug encapsulation capacity and rapid release [19,21].

The main objective of this study was to produce homogeneous and thermally stable hydrogels that can withstand body pH values (around 7) and temperatures (36-40 °C) without decomposing. In this way, chitosan was used as a raw material, and the influence of processing conditions such as agitation time, gelation temperature, pH and biopolymer concentration were evaluated to attain a full characterization of the properties of the systems. The optimization of the hydrogel formation was evaluated through rheological and microstructural characterization.

## 2. Materials and Methods

### 2.1. Materials

Low molecular weight chitosan (MW = 130,000 g/mol) with a deacetylation degree between 75 and 85% was provided by Sigma-Aldrich S.A. (Darmstadt, Germany). A 0.05 M solution of acetic acid was employed to dissolve chitosan, providing a pH of 3.2. In addition, a 4 M sodium hydroxide (NaOH) solution was employed to increase the pH of the hydrogels during gelation. Both acetic acid and NaOH were obtained from Panreac Química S.A. (Barcelona, Spain).

### 2.2. Hydrogels Production

The different hydrogels produced (Table 1) were processed by a complex coacervation method, following a protocol based on previous studies reported by Crompton et al. (2005), with the novelty of evaluating each of the parameters that affect hydrogel gelation separately in order to obtain the optimal parameters that lead to homogeneous hydrogels with neutral pH and thermal stability [22]. It is worth mentioning that Crompton et al. (2005) used 1.5 wt.% of chitosan and 50 °C for gelation temperature. For this, these parameters were used as references, changing them individually to evaluate their isolate behavior in the properties of the hydrogels. In this process, the biopolymer is primarily dissolved to improve chain mobility and favor their interconnection. This is usually achieved by agitation, which helps this process and is necessary to evaluate the agitation time. Regarding gelling, the pH change favors physical interactions, such as electrostatic interactions, hydrogen bonding, hydrophobic interactions as well as multi-physical ones [23]. Furthermore, the change from acidic to neutral pH value also generates protonation of amino groups, improving the strength of the formed hydrogel by electrostatic repulsions [24]. Lastly, as NaOH is incorporated superficially, gelation time and temperature are also two key aspects to take into account when evaluating pH diffusion and heterogeneity of the systems. With respect to this last parameter, Lavanya et al. (2020) state that temperature is related to hydrophobic constituents of the polymeric chains, which could be a key factor for the homogeneity of the hydrogel [25]. For this reason, agitation time, pH change and gelation temperature were selected as processing parameters. In addition, chitosan concentrations were evaluated in order to study their influence on the process.

First, 20 mL of each chitosan solution (1.0, 1.5 and 2.0 wt.% chitosan concentration) were prepared using 0.05 M acetic acid, reaching a pH value of 3.2. Then, the solutions were magnetically stirred at 50 °C for 1–2 h, depending on the system. These two stages were followed by a neutralization stage on some of the solutions where the pH was changed from 3.2 to 6.5–7 by adding 4 M NaOH with a sprayer to maintain the homogeneity of the sample (a constant pH value throughout the system). In this case, as gelation was superficially initiated by adding NaOH (pH change), 20–25 min were necessary to allow the complete gelation of the hydrogel, allowing the diffusion of the NaOH (no further changes were promoted for longer times) at a final temperature selected to aid the gelation process (4, 20 or 50 °C). When the hydrogel was not completely formed, only the gelled part was used for evaluation, indicating that these hydrogels were not homogeneous.

### 2.3. Hydrogels Characterization

#### 2.3.1. Rheological Characterization

The rheological characterization of the obtained hydrogels was carried out using an AR2000 rheometer (TA Instrument, New Castle, DE, USA) with a serrated plate-plate geometry (40 mm in diameter). This equipment was employed to perform four types of dynamic oscillatory shear tests:

Strain sweep tests: these measurements were carried out between 10^−4^ and 2% at a constant frequency of 1 Hz and at the final hydrogel temperature (4, 20 or 50 °C). In these tests, the lineal viscoelastic range (LVR) was determined, evaluating the critical strain (last strain in the LVR) of each system.

Frequency sweep tests: these tests were performed between 0.02 and 20 Hz at a constant strain (below the critical strain) and at the final hydrogel temperature. During these experiments, the elastic (G′) and viscous (G″) moduli were determined for the whole studied range. In addition, the elastic modulus, the loss tangent (tan δ = G″/G′) and the complex viscosity (|η*| = $\sqrt{{\left(G\prime \right)}^{2}+{\left(G\prime \prime \right)}^{2}}/\omega$) at 1 Hz (G′_1_, tan δ_1_ and ղ*_1_, respectively) of the systems were compared.

Time sweep tests: these measurements were carried out for 60 min to obtain the optimal gelation time of the different systems. The samples were subjected to constant stress at 1 Hz and a strain below the critical strain. Thus, G′ and G″ were evaluated over time.

Temperature cycle: a temperature ramp test was performed in the best hydrogel. The temperature was increased from 4 °C to 40 °C at a heating rate of 5 °C/min, a constant frequency of 1 Hz and a strain into the LVR. Then, the temperature was kept at 40 °C for 50 min to evaluate the stability of the hydrogel at this temperature.

#### 2.3.2. Microstructural Characterization

The microstructural characterization of the systems was carried out via scanning electron microscopy (SEM). Firstly, the samples were chemically fixed and dried to remove the water from the hydrogels without modifying their structure. Then, they were coated with a thin layer of palladium–gold. Finally, the samples were observed in a Zeiss EVO microscope (Pleasanton, CA, USA).

### 2.4. Statistical Analyses

A statistical study was carried out on each of the selected parameters. At least three replicates of each measure were carried out to calculate certain statistical parameters, using the Excel program, a Microsoft Office tool. In addition, an analysis of variance (ANOVA) was carried out with 95% confidence interval (*p* < 0.05) in order to determine whether the systems present significant differences with the SPSS 18 statistical package. For correlation coefficients and their significance, Pearson correlation coefficients and *p*-values were calculated. In this way, the parameters are presented as a mean value with its standard deviation, showing the significance of the values as superscripts, using different symbols when there are significant differences among the systems in the same parameter.

## 3. Results and Discussion

### 3.1. Study of the pH Change and Agitation Time

**Figure 1:**
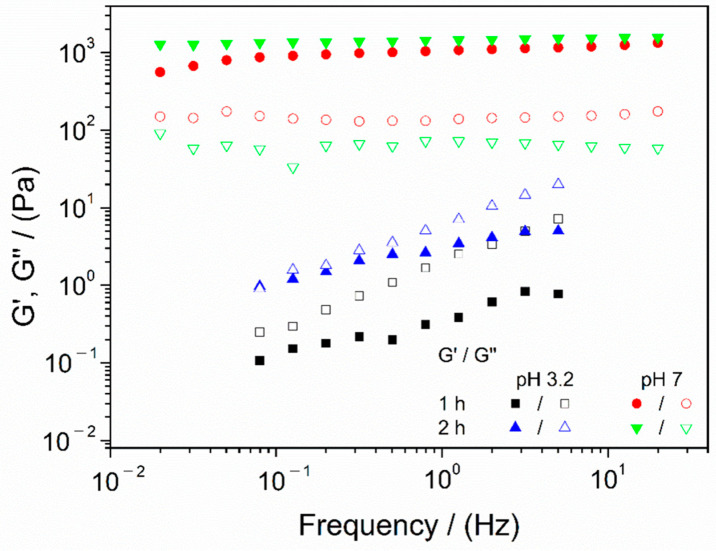
Frequency sweep tests of chitosan hydrogels processed at different agitation times (1 and 2 h) and without (pH 3.2) and with pH change (pH 7) gelatinized at 50 °C. The presented data are the mean values for each system.

As previously described, chitosan is a cationic polymer, which means that it has a positive net charge and is soluble at low pH values. Thus, polymer solutions were prepared at pH 3.2, using a 0.05 M acetic acid solution as a solvent. It is known that low pH values turn chitosan soluble, which means that higher values turn chitosan insoluble. Thus, an increment in the pH of the solution could lead to an improvement in the mechanical properties of the final hydrogel due to the protonation of amino groups. Figure 1 shows the frequency sweep tests of chitosan hydrogels processed with different conditions, namely, different agitation times (1 and 2 h), to evaluate if this parameter exerts any effect on the hydrogel final properties [22] and the application or not of a sudden pH increment to a pH value of approximately 7 (neutral pH), in order to modify the microstructure and to make it similar to the pH required for cell growth and proliferation [26].

The pH variation had a significant influence on the viscoelastic behavior of the hydrogel. In fact, neutral pH gels exhibited an elastic and strong gel behavior, with G′ (elastic modulus) values above G″ (viscous modulus) in the whole frequency range evaluated, with minimal variation of both moduli with frequency. In contrast, systems that maintained acidic pH showed a great dependence of both moduli on the frequency and, as can be observed, G″ values were higher than those obtained for G′, which means that these systems were concentrated solutions, instead of hydrogels, since they did not show a solid character. This was already predicted by Li et al. (2020), which indicates that acidic pH values generate certain stability in cationic hydrogels, being favored at higher pH values, since these groups are protonated, reinforcing the structure [24].

On the other hand, the results obtained for different agitation times revealed that the systems agitated for one or two hours are highly similar; thus, it can be stated that this parameter does not exert a significant effect on the properties of the final hydrogels. Therefore, the minimal agitation time (1 h) was selected to optimize the process.

For a better comparison of the properties obtained for these systems, several parameters are gathered in Table 2, namely, critical strain, elastic modulus, complex viscosity and loss tangent at 1 Hz (G′_1_, η*_1_ and tan(δ)_1_, respectively).

**Figure 2:**
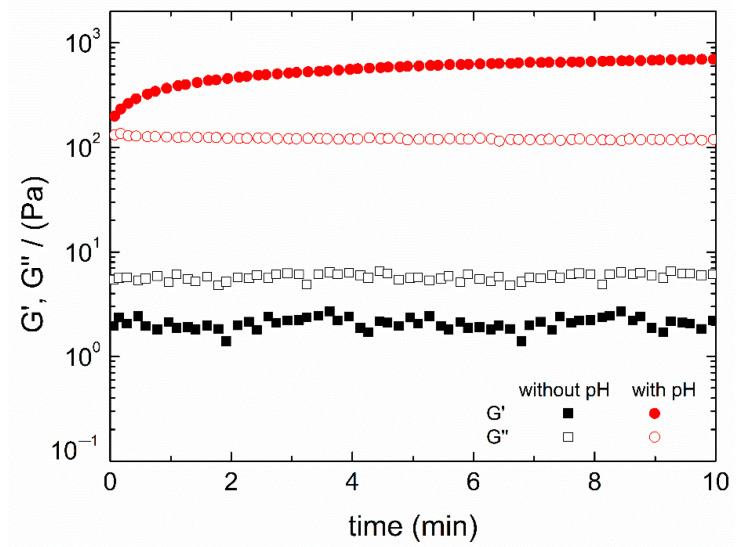
Time tests and macroscopic aspect of chitosan hydrogels processed without and with pH change gelatinized at 50 °C. The presented data are the mean values for each system.

The results gathered in Table 2 confirmed that hydrogels with pH variation were stiffer (higher elastic modulus, viscosity and lower loss tangent) and, consequently, less deformable. Moreover, the loss tangent values obtained for hydrogels with pH change (below 0.15 in both cases) proved the strong gel behavior previously mentioned. Therefore, the application of pH change from acidic to neutral pH was the key factor that enabled the gelation process. In order to confirm this statement, time tests were carried out, obtaining the results shown in Figure 2, together with the macroscopic aspect of both systems (with and without pH change).

As can be observed, when the pH was changed, G′ values were above G″ ones, which means that, as previously described, the solution turns into a hydrogel type structure, whereas when the pH was maintained and no change was applied, G″ values were higher than G′ values, maintaining a concentrated solution behavior. Thus, no gelation was observed for constant pH. This can also be observed in the macroscopic images, where certain gelation is appreciable when a pH change was applied (although in a heterogeneous way) while it remained as a transparent viscous fluid when the pH was maintained at acidic values.

On the other hand, it seems that, as previously analyzed, agitation time had no influence on the behavior of the evaluated hydrogels for more than 1 h, as can be observed in the values in Table 2, since there are no significant variations between the systems agitated for 1 or 2 h. Consequently, the application of pH change and 1 h of agitation time were the selected parameters to optimize the fabrication process.

### 3.2. Study of the Gelation Temperature

The previous section demonstrated that chitosan requires a modification in its pH to form a hydrogel. Some authors assert that when pH is nearly 7, the gelation is favored at low temperatures, whereas when exposed to higher temperatures, the solution gelatinizes at lower pH [27]. Therefore, in order to verify the effect of temperature on the gelation process, three different gelation temperatures (4, 20 and 50 °C) were studied.

**Figure 3:**
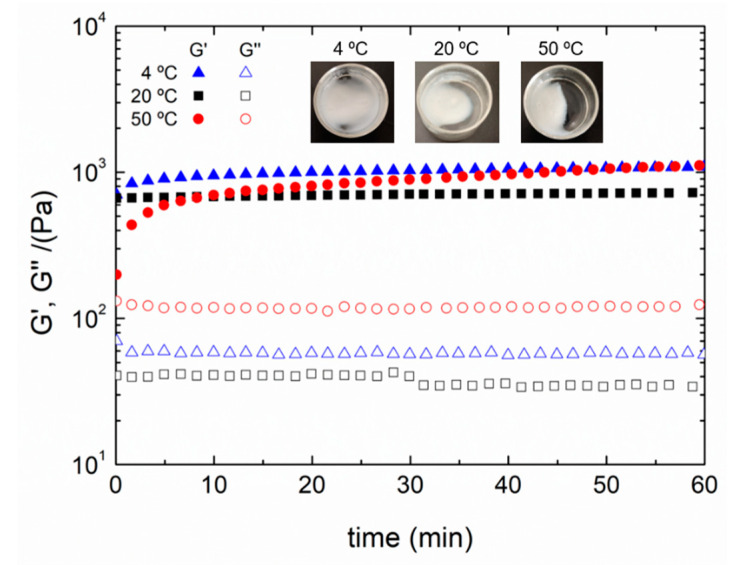
Time tests and macroscopic aspects of chitosan hydrogels processed at different gelation temperatures (4, 20 and 50 °C). The presented data are the mean values for each system.

Figure 3 shows the results of time sweep tests of chitosan hydrogels processed at the different gelation temperatures, as well as the macroscopic aspect of the respective hydrogels.

**Figure 4:**
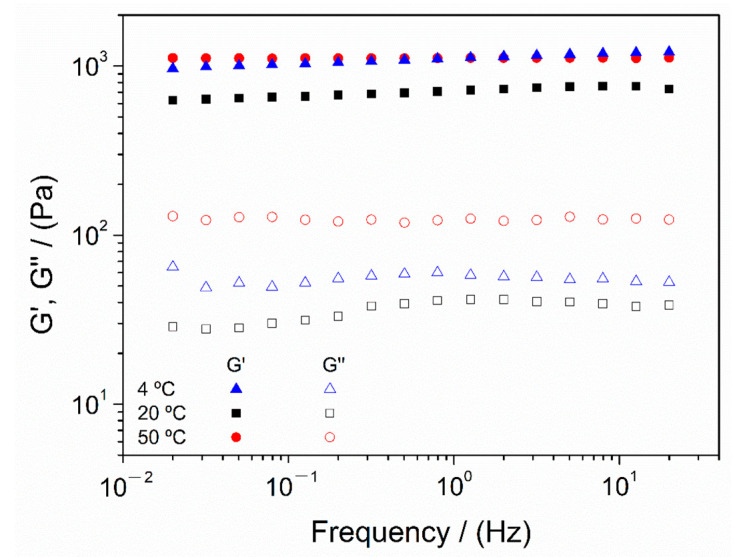
Frequency sweep tests of chitosan hydrogels processed at different gelation temperatures (4, 20 and 50 °C). The presented data are the mean values for each system.

In all cases, a gel behavior can be observed, with G′ values above G″ ones and with barely any modifications during the test time (only a slight increase). Comparing the results obtained for each system, it can be noted that the hydrogel processed with a gelation temperature of 50 °C is not as strong as the other gels, since the relation between G′ and G″ is weaker than for the rest of the systems. This is probably due to the heterogeneity observed in this hydrogel (macroscopic aspect). For the hydrogels processed at 4 °C and 20 °C gelation temperature, it can be observed that the former is slightly stiffer than the latter. In fact, regarding the macroscopic aspect of each hydrogel, the one processed at a lower temperature presented a more homogeneous structure than the others and thus, increasing gelation temperature leads to a more heterogeneous structure. This behavior can also be proved by conducting frequency sweep tests for each hydrogel, whose results are represented in Figure 4.

All systems displayed a similar strong gel behavior, without significant variations of moduli with frequency and the elastic modulus (G′) was significantly higher than the viscous one (G″). In any case, it seems that, when comparing every hydrogel, there are differences among moduli values between each system depending on the gelation temperature. Therefore, in order to establish a better comparison, selected mechanical properties are gathered in Table 3.

The results showed that the hydrogel processed at 4 °C exhibited improved mechanical properties, with better stiffness (higher G′_1_ and lower tan (δ)_1_). On the other hand, although the hydrogel produced at 50 °C exhibited similar stiffness and higher viscosity, it can be observed that the value obtained for critical strain is especially lower than that obtained for the other systems. This could be attributed to the heterogeneity of the system, which can be observed in the macroscopic images of the hydrogel (Figure 3). In this way, although the higher temperature favors chain mobility, as well as pH diffusion, the induced heterogeneity leads to a higher relationship between elastic and viscous modulus (tan (δ)) compared to other systems, producing a less strong hydrogel. Therefore, despite having similar mechanical properties as the hydrogel processed at 50 °C, 4 °C was selected as the best gelation temperature due to its better critical strain and homogeneity.

### 3.3. Study of Chitosan Concentration

**Figure 5:**
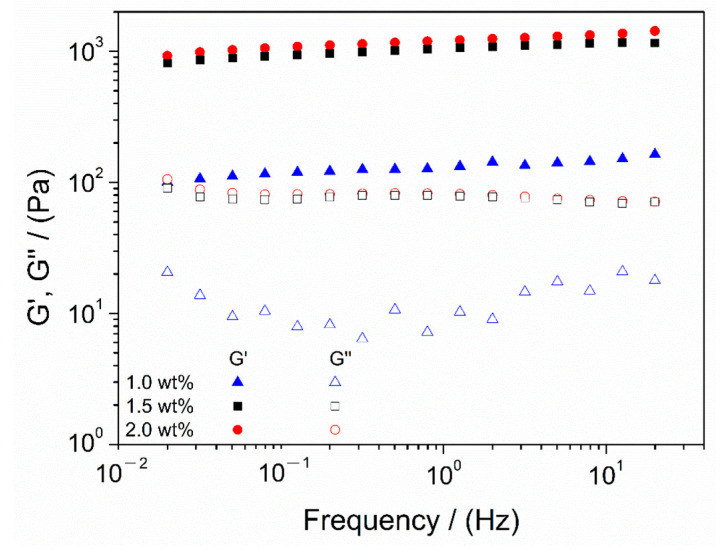
Frequency sweep tests of chitosan hydrogels with different biopolymer concentrations (1.0, 1.5 and 2.0 wt.%) gelatinized at 4 °C. The presented data are the mean values for each system.

Once the optimal processing conditions were established, namely, 1 h agitation time for the solution at pH 3.2, subsequently increasing the pH to 7 and ending with a gelation temperature of 4 °C, the next parameter to be evaluated was chitosan concentration. Thus, hydrogels with different chitosan concentrations (1.0, 1.5 and 2.0 wt.%) were prepared in order to assess the effect of this parameter. Figure 5 depicts the results obtained from frequency sweep tests of chitosan hydrogels with different biopolymer concentrations.

Every system displayed a stable behavior with insignificant variation with frequency, especially the 1.5 and 2.0 wt.% systems, and elastic behavior, as G′ values were above G″ values in the whole frequency range evaluated. It can be observed that hydrogels with chitosan concentrations of 1.5 and 2.0 wt.% had very similar behavior, whereas the system with 1.0 wt.% proved to be very different, as it was softer (lower G′ and G″ values) than the others. Therefore, higher concentrations of chitosan led to better mechanical properties. Nevertheless, at a certain biopolymer concentration, there are no significant variations observed in the mechanical properties, reaching a maximum. For a better comparison, the values of critical strain, elastic modulus, complex viscosity and loss tangent at 1 Hz obtained for each system are gathered in Table 4.

As previously observed, the mechanical properties improved for increasing chitosan concentration. Higher values (over one order of magnitude) of critical strain, elastic modulus and complex viscosity were observed for higher concentrations (1.5 and 2.0 wt.%), compared to the values obtained for the hydrogel with 1.0 wt.% of chitosan. Nevertheless, no significant changes were detected for loss tangent, which, along with their low values, denoted a strong gel-like behavior. Furthermore, it should be noted that there were no significant variations between the systems, with higher concentrations indicating a constant tendency for rheological properties when the biopolymer concentration is increased, as described by other authors [28].

Therefore, the analysis of the mechanical properties and processing conditions of chitosan hydrogels revealed that the best system for its application in TE was a hydrogel with 1.5 wt.% of chitosan, agitated for 1 h at pH 3.2, then neutralized to 7 and with a final gelation temperature of 4 °C.

### 3.4. Microstructure Evaluation

**Figure 6:**
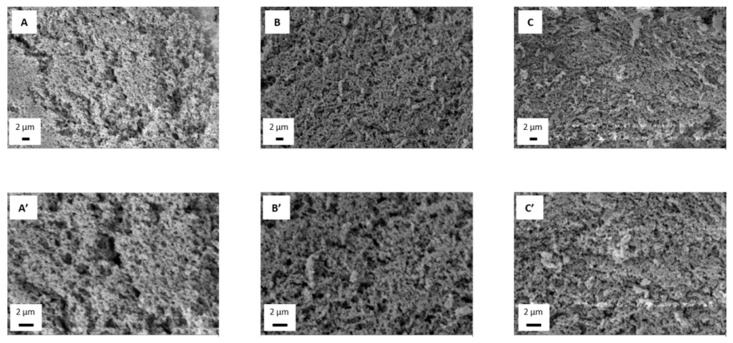
Microstructure of chitosan hydrogels with different biopolymer concentrations (1.0 (**A**,**A’**), 1.5 (**B**,**B’**) and 2.0 (**C**,**C’**) wt.%) gelated at 4 °C.

A microstructural evaluation of the hydrogels was carried out once the optimal processing conditions were selected and the mechanical properties were evaluated. This characterization allows establishing a relationship between the structure of hydrogel systems and the variation in their properties with chitosan concentration. Thus, hydrogels with 1.0, 1.5 and 2.0 wt.% of chitosan were characterized by SEM, obtaining the resulting mages depicted in Figure 6.

The images show a similar sponge-like structure with a homogeneous pore distribution in all cases. All of them similar to the microstructure obtained by other authors in chitosan hydrogels processed by different protocols [29,30]. However, although the pores are homogeneously distributed, it can be observed that the pore size is different in each hydrogel, being larger for the lowest concentration (A and A’) and progressively decreasing in size with the increment of chitosan concentration, leading to a structure with smaller pores for 2.0 wt.% hydrogel (C and C’). Greater chitosan concentration implies more biopolymer chains, which favors crosslinking between chains and, thus, reducing pore size. This explains the larger pores observed in the 1.0 wt.% hydrogel and then the 1.5 wt.% hydrogel (B and B’) shows similar-sized pores, but also smaller ones, indicating the beginning of the size reduction due to the increment in chitosan concentration, obtaining the smallest pores for the 2.0 wt.% hydrogel (C and C’).

These results are in line with the previous mechanical evaluation of the hydrogels, since it was observed that the hydrogel with 1.0 wt.% chitosan concentration had worse mechanical properties compared with the other systems, which is due to its microstructure. As it has larger pores than the rest, its structure is filled with more water, making its mechanical behavior more irregular. Increasing chitosan concentration led to more consistent microstructures, with smaller pores, then resulting in better mechanical properties, as observed, although no significant differences were appreciated between the 1.5 and 2.0 wt.%.

### 3.5. Temperature Study

**Figure 7:**
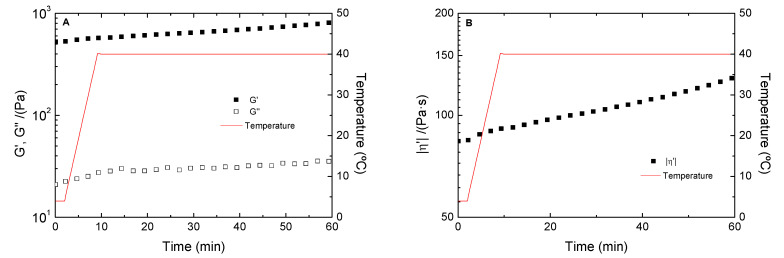
Temperature ramp tests of chitosan hydrogels processed at 4 °C and 1.5 wt.% chitosan. The presented data are the mean values for each system. (**A**) Elastic (G′) and viscous (G″) moduli profile. (**B**) Complex viscosity profile.

Finally, the thermal stability of the chosen hydrogel was evaluated by time sweep tests (Figure 7). As can be seen, the hydrogels are highly stable, since G′ and G″ are independent of time, even when the temperature was increased to 40 °C. In addition, complex viscosity (Figure 7B) increases smoothly when the temperature changed from 4 to 40 °C, which could be due to an increase in the hydrogel crosslinking degree. In any case, this hydrogel is stable enough to be used as a biomaterial because it is resistant to the bioreactor temperature, where cells are grown, and to the temperature of the human body, where they will proliferate to regenerate tissues.

## 4. Conclusions

To sum up, it can be concluded that chitosan is a suitable raw material to process homogeneous and thermally stable hydrogels that can be used as biomaterials in regenerative medicine applications. In this way, a pH change from 3.2 to 7 proved to be essential for the formation of the hydrogel. In addition, although the agitation time does not seem to have an implication on the properties of the hydrogel, the gelation temperature and the concentration of the biopolymer alter its properties. In this way, the optimal conditions to obtain homogeneous, thermally stable and mechanically and morphologically adequate hydrogels are an agitation time of 1 h with pH change, a gelation temperature of 4 °C and a chitosan concentration of 1.5 wt.%. Nevertheless, it is necessary, in future studies, to evaluate the biological properties of these hydrogels before their use as biomaterials.

## Figures and Tables

**Table 1:** Studied systems obtained for different processing conditions.

System	Chitosan Concentration (wt.%)	Agitation Time (h)	pH Change	Final Temperature (°C)
1	1.5	1	Yes	50
2	1.5	1	No	50
3	1.5	2	Yes	50
4	1.5	2	No	50
5	1.5	1	Yes	20
6	1.5	1	Yes	4
7	1.0	1	Yes	4
8	2.0	1	Yes	4

**Table 2:** Strain and frequency sweep tests parameters (mean value + standard deviation) for chitosan hydrogels processed at different agitation times (1 and 2 h) and without and with pH change gelatinized at 50 °C. G′_1_: elastic modulus at 1 Hz; tan δ_1_: loss tangent at 1 Hz; η*_1_: complex viscosity at 1 Hz.

Systems		Critical Strain (%)	G′_1_ (Pa)	tan(δ)_1_ (-)	η*_1_ (Pa·s)
Agitation Time	pH Change				
1 h	No	1.01 ± 0.05 ^I^	3.47 ± 0.42 ^a^	2.04 ± 0.03 ^A^	0.99 ± 0.26 ^α^
	Yes	0.48 ± 0.08 ^II^	1117 ± 12 ^b^	0.11 ± 0.01 ^B^	184 ± 29 ^β^
2 h	No	0.42 ± 0.08 ^II^	0.38 ± 0.07 ^c^	2.93 ± 0.05 ^C^	0.57 ± 0.17 ^α^
	Yes	0.48 ± 0.11 ^II^	1456 ± 22 ^d^	0.04 ± 0.01 ^D^	191 ± 43 ^β^

Different symbols, included as superscripts (I, II; a, b, c, d; A, B, C, D; α, β), indicate significant differences (*p* < 0.05) in each column.

**Table 3:** Strain and frequency sweep tests parameters (mean value + standard deviation) for chitosan hydrogels processed at different gelation temperatures (4, 20 and 50 °C). G′_1_: elastic modulus at 1 Hz; tan δ_1_: loss tangent at 1 Hz; η*_1_: complex viscosity at 1 Hz.

Gelation Temperature	Critical Strain (%)	G′_1_ (Pa)	tan(δ)_1_ (-)	η*_1_ (Pa·s)
4 °C	1.01 ± 0.11 ^I^	1122 ± 15 ^a^	0.05 ± 0.02 ^A^	142 ± 17 ^α^
20 °C	1.01 ± 0.05 ^I^	720 ± 12 ^b^	0.06 ± 0.03 ^A^	91 ± 16 ^β^
50 °C	0.48 ± 0.08 ^II^	1117 ± 12 ^a^	0.11 ± 0.01 ^B^	184 ± 29 ^γ^

Different symbols, included as superscripts (I, II; a, b; A, B; α, β, γ), indicate significant differences (*p* < 0.05) in each column.

**Table 4:** Strain and frequency sweep tests parameters (mean value + standard deviation) for chitosan hydrogels with different biopolymer concentrations (1.0, 1.5 and 2.0 wt.%) gelatinized at 4 °C. G′_1_: elastic modulus at 1 Hz; tan δ_1_: loss tangent at 1 Hz; η*_1_: complex viscosity at 1 Hz.

Biopolymer Concentration	Critical Strain (%)	G′_1_ (Pa)	tan(δ)_1_ (-)	η*_1_ (Pa·s)
1.0 wt.%	0.48 ± 0.08 ^I^	130 ± 12 ^a^	0.08 ± 0.01 ^A^	21 ± 6 ^α^
1.5 wt.%	1.01 ± 0.11 ^II^	1122 ± 15 ^b^	0.05 ± 0.02 ^A^	142 ± 17 ^β^
2.0 wt.%	1.01 ± 0.11 ^II^	1049 ± 97 ^b^	0.06 ± 0.04 ^A^	201 ± 21 ^γ^

Different symbols, included as superscripts (I, II; a, b; A; α, β, γ), indicate significant differences (*p* < 0.05) in each column.

## Data Availability

The data presented in this study are available on request from the corresponding author.

## References

[1] Schultheiss D., Bloom D.A., Wefer J., Jonas U. (2000). Tissue engineering from Adam to the zygote: Historical reflections. World J. Urol..

[2] Liu C., Xia Z., Czernuszka J.T. (2007). Design and development of three-dimensional scaffolds for tissue engineering. Chem. Eng. Res. Des..

[3] Deo K.A., Lokhande G., Gaharwar A.K. (2019). Nanostructured Hydrogels for Tissue Engineering and Regenerative Medicine.

[4] Hoffman A.S. (2012). Hydrogels for biomedical applications. Adv. Drug Deliv. Rev..

[5] Zhu L., Liu Y., Jiang Z., Sakai E., Qiu J., Zhu P. (2019). Highly temperature resistant cellulose nanofiber/polyvinyl alcohol hydrogel using aldehyde cellulose nanofiber as cross-linker. Cellulose.

[6] Dhandayuthapani B., Yoshida Y., Maekawa T., Kumar D.S. (2011). Polymeric Scaffolds in Tissue Engineering Application: A Review. Int. J. Polym. Sci..

[7] Celikkin N., Rinoldi C., Costantini M., Trombetta M., Rainer A., Święszkowski W. (2017). Naturally derived proteins and glycosaminoglycan scaffolds for tissue engineering applications. Mater. Sci. Eng. C.

[8] Tariverdian T., Sefat F., Gelinsky M., Mozafari M., Mozafari M., Sefat F., Atala A. (2019). 10—Scaffold for bone tissue engineering. Woodhead Publishing Series in Biomaterials.

[9] Kim M., Tang S., Olsen B.D. (2013). Physics of engineered protein hydrogels. J. Polym. Sci. Part B Polym. Phys..

[10] Van Vlierberghe S., Dubruel P., Schacht E. (2011). Biopolymer-Based Hydrogels as Scaffolds for Tissue Engineering Applications: A Review. Biomacromolecules.

[11] Rao M.G., Bharathi P., Akila R. (2014). A comprehensive review on biopolymers. Sci. Rev. Chem. Commun..

[12] Rebelo R., Fernandes M., Fangueiro R. (2017). Biopolymers in Medical Implants: A Brief Review. Procedia Eng..

[13] Hannibal-Paci C. (1995). Information to Users Umi. Ph.D. Thesis.

[14] Khan F., Ahmad S.R. (2013). Polysaccharides and Their Derivatives for Versatile Tissue Engineering Application. Macromol. Biosci..

[15] Chakraborty M., Ghosh A., Ghosh U.U., DasGupta S. Enhanced cooling by an oscillating droplet on DMF platform. Proceedings of the 2015 AIChE Annual Meeting.

[16] Rodríguez-Pedroso A.T., Ramírez-Arrebato M.A., Rivero-González D., Bosquez-Molina E., Barrera-Necha L.L., Bautista-Baños S. (2009). Propiedades Químico-Estructurales Y Actividad Biológica De La Quitosana. Rev. Chapingo Ser. Hortic..

[17] Hu J., Andablo-Reyes E., Soltanahmadi S., Sarkar A. (2020). Synergistic Microgel-Reinforced Hydrogels as High-Performance Lubricants. ACS Macro Lett..

[18] Ding F., Zou Y., Wu S., Zou X. (2020). Self-healing and tough hydrogels with conductive properties prepared through an interpenetrating polymer network strategy. Polymer.

[19] Tsirigotis-Maniecka M., Szyk-Warszyńska L., Maniecki Ł., Szczęsna W., Warszyński P., Wilk K.A. (2021). Tailoring the composition of hydrogel particles for the controlled delivery of phytopharmaceuticals. Eur. Polym. J..

[20] Rivero Berti I., Islan G.A., Castro G.R. (2021). Enzymes and biopolymers. The opportunity for the smart design of molecular delivery systems. Bioresour. Technol..

[21] Daud H., Ghani A., Iqbal D.N., Ahmad N., Nazir S., Muhammad M.J., Hussain E.A., Nazir A., Iqbal M. (2021). Preparation and characterization of guar gum based biopolymeric hydrogels for controlled release of antihypertensive drug. Arab. J. Chem..

[22] Crompton K.E., Prankerd R.J., Paganin D.M., Scott T.F., Horne M.K., Finkelstein D.I., Gross K.A., Forsythe J.S. (2005). Morphology and gelation of thermosensitive chitosan hydrogels. Biophys. Chem..

[23] Fu J., Yang F., Guo Z. (2018). The chitosan hydrogels: From structure to function. New J. Chem..

[24] Li C.-P., Weng M.-C., Huang S.-L. (2020). Preparation and Characterization of pH Sensitive Chitosan/3-Glycidyloxypropyl Trimethoxysilane (GPTMS) Hydrogels by Sol-Gel Method. Polymers.

[25] Lavanya K., Chandran S.V., Balagangadharan K., Selvamurugan N. (2020). Temperature- and pH-responsive chitosan-based injectable hydrogels for bone tissue engineering. Mater. Sci. Eng. C.

[26] Ahmad A.M. (2007). Potential pharmacokinetic interactions between antiretrovirals and medicinal plants used as complementary and African traditional medicines. Biopharm. Drug Dispos..

[27] Bhattarai N., Gunn J., Zhang M. (2010). Chitosan-based hydrogels for controlled, localized drug delivery. Adv. Drug Deliv. Rev..

[28] Calero N., Muñoz J., Ramírez P., Guerrero A. (2010). Flow behaviour, linear viscoelasticity and surface properties of chitosan aqueous solutions. Food Hydrocoll..

[29] Milosavljević N.B., Ristić M.Đ., Perić-Grujić A.A., Filipović J.M., Štrbac S.B., Rakočević Z.L., Krušić M.T.K. (2011). Removal of Cu^2+^ ions using hydrogels of chitosan, itaconic and methacrylic acid: FTIR, SEM/EDX, AFM, kinetic and equilibrium study. Colloids Surf. A Physicochem. Eng. Asp..

[30] Huang B., Liu M., Zhou C. (2017). Chitosan composite hydrogels reinforced with natural clay nanotubes. Carbohydr. Polym..

